# Diabetic retinopathy is associated with pulse wave velocity, not with the augmentation index of pulse waveform

**DOI:** 10.1186/1475-2840-7-11

**Published:** 2008-04-25

**Authors:** Osamu Ogawa, Kiyoko Hiraoka, Takahiro Watanabe, Junichiro Kinoshita, Masahiko Kawasumi, Hidenori Yoshii, Ryuzo Kawamori

**Affiliations:** 1 Midorigaoka Ekimae Clinic, 1-1-1, Midorigaoka, Yachiyo city, Chiba Pref., 276-0049, Japan; 2 Department of Medicine, Metabolism, and Endocrinology, Juntendo University School of Medicine, 2-1-1 Hongo, Bunkyo-ku, Tokyo 113-8421, Japan

## Abstract

**Background:**

To investigate the clinical differences between pulse wave velocity and augmentation index in diabetic retinopathy.

**Methods:**

The subjects were 201 patients with type 2 diabetes. These subjects were measured for both augmentation index (AI) and brachial-ankle pulse wave velocity (baPWV) by a pulse wave analyzer. The relationships between AI, baPWV, and diabetic retinopathy were examined.

**Results:**

BaPWV was significantly higher in patients with diabetic retinopathy than in individuals without the disease. (20.13 ± 3.66 vs.17.14 ± 3.60 m/s p < 0.001) AI was higher in patients with diabetic retinopathy, but not significantly. (19.5 ± 15.2 vs. 14.8 ± 20.5% p = 0.14) The association between baPWV and diabetic retinopathy remained statistically significant after adjustment. (Odds ratio: 1.21 Per m/s, 95% confidence interval: 1.07–1.37) On the other hand, the association between AI and diabetic retinopathy was not statistically significant. (Odds ratio: 1.01 Per %, 95% confidence interval: 0.98–1.03)

**Conclusion:**

BaPWV is associated with diabetic retinopathy, but AI is not. The clinical significance appears to be different between PWV and AI in patients with diabetes.

## Background

Both pulse wave velocity (PWV) and augmentation index (AI) are known as markers of arterial stiffness [1]. These parameters have been associated with cardiovascular disease. The definition and measurement of these parameters is different, therefore, the clinical significance may also be different. PWV was defined as the speed with which the pulse wave travels along the length of an artery. Augmentation of the pulse waveform was defined as the difference between the second systolic peak (caused by wave reflection) and the first systolic peak (caused by left ventricular ejection). AI was this difference expressed as a percentage of the central pulse pressure. It is reported that type 2 diabetes is associated with increased AI, and decreased carotid-femoral transit time, which is related to PWV [2]. On the other hand, it has been reported that there is no association between AI and PWV in patients with diabetes [3]. Thus, the clinical difference between PWV and AI remains uncertain in patients with diabetes. We previously reported an association between diabetic retinopathy and brachial-ankle pulse wave velocity (baPWV) [4]. However, we did not investigate the relationship between AI and diabetic retinopathy. In this study, to clarify the clinical difference between PWV and AI, we investigated the association between baPWV, AI and diabetic retinopathy.

## Methods

Japanese patients with type 2 diabetes who had randomly undergone testing for both baPWV and AI of the common carotid artery between April 2002 and April 2003 at the outpatient department of the Juntendo University Hospital (Tokyo, Japan) were enrolled in this study. Informed consent was obtained from all subjects. Clinical data were collected in a cross sectional manner from medical records. Type 2 diabetes was indicated by a fasting plasma glucose concentration >7.0 mmol/L without treatment, a plasma glucose concentration 11.0 mmol/L measured 2 h after a 75 g oral glucose load, or by treatment with a glucose-lowering drug. The patients selected for the study were in a state of sinus rhythm. The patients selected for the study had no history of ketoacidosis, ischemic heart disease, peripheral artery disease or ischemic stroke. Patients with an ankle-brachial pressure index (ABI) <0.9 were excluded from the analysis. Diagnoses of diabetic retinopathy were made by ophthalmologists based on the presence of one or more of the following clinical features in the fundus: hemorrhages, hard or soft exudate, venous beading, intra-retinal microvascular abnormalities, cotton-wool spots, pre-retinal new vessels, fibrous proliferation and/or photocoagulation scars. Diabetic nephropathy was defined as a casual urinary albumin/creatinine ratio >30 mg/g Cr. Total cholesterol, high-density lipoprotein (HDL) cholesterol, triglyceride and hemoglobin A1c (HbA1c), urinary albumin and creatinine levels were determined using standard laboratory techniques.

### Measurements of PWV and AI

Measurement of baPWV and AI were performed on the same day. The automatic pulse wave analyzer (Form/ABI with AT-unit; Omron Colin Co. Ltd, Tokyo) was used to measure baPWV, AI and ABI. This device has been approved by the US food and drug administration. The device simultaneously performs an electrocardiogram, phonocardiogram, bilateral brachial and ankle blood pressure (BP), and carotid arterial pulse wave. The detailed method to measure baPWV and ABI was previously reported [5,6]. Briefly, measurement was performed with the patients in the supine position after resting for five minutes. Occlusion and monitoring cuffs were placed snugly around sites in the upper and lower extremities. Pressure waveforms were then recorded simultaneously from the brachial arteries by an oscillometric method. The ABI was calculated as follows: ABI = ankle systolic BP/brachial systolic BP. It is well known that patients with a low ABI exhibit a lower baPWV value than the actual value when assessed by the method described above [7]. Thus, we excluded patients with an ABI level of less than 0.9 when evaluating baPWV.

Carotid arterial pulse wave were obtained using arterial applanation tonometry incorporating an array of 15 micropiezoresistive transducers. Advanced multiplexing electronics combined with automatic sensor selection relative to the target artery facilitated and ensured optional placement of the sensor. The carotid sensor was manually placed on the common carotid artery. The systolic portion of the central arterial waveform is characterized by two pressure peaks. The first peak is caused by left ventricular ejection, whereas the second peak is a result of wave reflection. The difference between both pressure peaks reflects the degree to which central arterial pressure is augmented by wave reflection. The AI (%) is defined as the percentage of the central pulse pressure which is represented by the reflected pulse wave and, therefore, reflects the degree to which central arterial pressure is augmented by wave reflection [8]. The reliability and reproducibility of the AI measured by this device are reported to be very good [9,10]. These measurements were taken with patients lying in a supine position after 5 min of rest.

### Statistical analysis

In order to compare the association between baPWV and diabetic retinopathy, and between AI and diabetic retinopathy, we performed several statistical analyses. First, subjects were divided into two groups: those with and those without diabetic retinopathy. Clinical characteristics, including baPWV and AI, were analyzed within the two groups. Continuous variables were presented as the mean standard deviation (S.D.). Continuous variables were compared using Student's t-test and categorical data were compared using the chi-squared test. Second, we used linear regression analysis to investigate the correlation between baPWV and AI. Third, multiple regression analysis was performed to investigate the relationship between arterial stiffness markers and clinical parameters. Multiple logistic-regression analysis was used to provide the adjusted odds ratios (ORs) for baPWV and AI for diabetic retinopathy. The following factors were adjusted for: age, gender, duration of diabetes, HbA1c level, diabetic nephropathy, hypertension (systolic BP > 140 mmHg and/or diastolic BP > 90 mmHg) and hyperlipidemia (total cholesterol concentration > 5.7 mmol/L and/or triglyceride concentration >1.7 mmol/L and/or treatment with antihyperlipidemic agents). In addition, AI was increasing more in younger individuals [11,12]. Therefore, the subjects were divided into two groups, under 60 years old and over 60 years old. We analyzed relationship between AI and retinopathy each groups. The ORs are presented with 95% confidence intervals (CIs). All statistical analyses were performed using StatView version 5.0 computer software (SAS Institute Inc. Cary, NC, USA). Probability values (p) < 0.05 were considered statistically significant.

## Results

The presence of diabetic retinopathy was confirmed in 47 (23.4%) of the 201 patients who were eligible to take part in the study. Table 1 shows the characteristics of the subjects according to the presence or absence of diabetic retinopathy. BaPWV, duration of diabetes, body height, heart rate, systolic BP and total cholesterol were all significantly higher in patients with diabetic retinopathy than in individuals without the disease. The proportion of female patients, presence of nephropathy and use of antihypertensive agents were also higher among those with diabetic retinopathy. AI was higher in patients with diabetic retinopathy, but not statistically significant. On linear regression analysis, the correlation between baPWV and AI was weak (r = 0.19, p = 0.007).

**Table 1:** Characteristics of the 201 patients enrolled in the study according to the presence (+) or absence (-) of diabetic retinopathy.

	DR(+) n = 47	DR(-) n = 154	P value
baPWV (m/s)	20.13 ± 3.66	17.14 ± 3.60	<0.001
AI (%)	19.5 ± 15.2	14.8 ± 20.4	0.14
Height (m)	1.59 ± 0.08	1.63 ± 0.08	0.003
Age (years)	61.1 ± 10.6	59.7 ± 11.2	0.44
Men (n)	25 (53%)	110 (71%)	0.03
Duration (years)	13.0 ± 8.5	8.6 ± 7.5	<0.001
Systolic BP (mmHg)	142.7 ± 16.3	129.3 ± 17.9	<0.001
Diastolic BP (mmHg)	82.8 ± 11.1	78.3 ± 9.56	0.007
Heart rate (beat/min)	72.4 ± 12.0	66.3 ± 10.5	0.001
HbA1c (%)	7.2 ± 1.5	7.4 ± 1.9	0.56
Total cholesterol (mmol/L)	5.88 ± 2.74	5.14 ± 0.89	0.004
HDL cholesterol (mmol/L)	1.40 ± 0.47	1.31 ± 0.40	0.22
Triglycerides (mmol/L)	1.68 ± 0.88	1.74 ± 1.12	0.75
Nephropathy	27 (18%)	27 (57%)	<0.001
Antihypertensive agents (yes/no)	129/150	251/629	<0.001

Mean values are indicated ± the standard deviation. DR, diabetic retinopathy.

On the multiple regression analysis, age, duration, heart rate, and SBP were dependent determinants of baPWV, while age, height, heart rate, and DBP were independent determinants of AI. (table 2)

**Table 2:** multiple regression analysis of baPWV and AI

	BaPWV		AI	
	r	p	r	p
Age	0.37	<0.001	0.29	<0.001
Height	-0.04	0.50	-0.31	<0.001
SBP	0.50	<0.001	0.02	0.86
DBP	-0.16	0.06	0.26	0.01
Heart rate	0.25	<0.001	-0.40	<0.001
Duration	0.12	0.02	-0.03	0.61
HbA1c	0.02	0.74	-0.05	0.41

Correlation coefficients of multiple regression (r) and the level of significance are shown.

The results of the logistic regression analyses are shown in table 3. The association between baPWV and diabetic retinopathy remained statistically significant after adjustment. On the other hand, the association between AI and diabetic retinopathy was not statistically significant. Duration, diabetic nephropathy were also significantly associated with diabetic retinopathy after adjustment. ORs of AI in group over 60 years old was 1.00 (95%CI 0.97–1.04), odds ratio of AI in group under 60 years old was 1.00 (95%CI 0.97–1.05) after adjusted age, gender, duration of diabetes, HbA1c, hypertension, hyperlipidemia, nephropathy.

**Table 3:** Adjusted odds ratios for diabetic retinopathy

Independent variable		Adjusted odds ratio (95% CI)	p	Adjusted odds ratio (95% CI)	p
Age	Per year	0.95(0.91–0.99)	0.02	0.97(0.94–1.05)	0.21
Sex	Female	1.00		1.00	
	Male	0.33 (0.14–0.78)	0.01	0.37 (0.16–0.88)	0.02
Duration of diabetes	(per year)	1.06 (1.004–1.11)	0.03	1.07 (1.02–1.12)	0.01
HbA1c	Per %	0.96(0.75–1.21)	0.7	0.97(0.77–1.21)	0.75
Hypertension	No	1.00		1.00	
	Yes	1.37 (0.59–3.19)	0.47	1.91 (0.86–4.28)	0.11
Hyperlipidemia	no	1.00		1.00	
	yes	0.96 (0.42–2.18)	0.91	0.90 (0.41–1.99)	0.80
Diabetic nephropathy	No	1.00		1.00	
	yes	4.57 (2.04–10.28)	<0.001	5.24 (2.40–11.4)	<0.001
baPWV	Per m/s	1.21(1.07–1.37)	0.002	-	
AI	Per %	-		1.01(0.98–1.03)	0.65

Diabetic retinopathy was used as dependent variable., CI: confidence interval

## Discussion

In this study, there was a significant positive association between the presence of diabetic retinopathy and baPWV among Japanese type 2 diabetic patients without macroangiopathy. However, the association between AI and diabetic retinopathy was found to be uncertain. The correlation between baPWV and AI was weak. The determining factors were different between baPWV and AI.

Previously, we showed baPWV is associated with diabetic retinopathy [4]. PWV is correlated with the duration of diabetes and with the accumulation of fluorescent advanced glycation end products in the aorta[13]. The accumulation of advanced glycation end products has also been associated with the progression of diabetic retinopathy [14,15]. Furthermore, the United Kingdom Prospective Diabetes Study (UKPDS) showed that the development of retinopathy was associated with long-term glycemic exposure [16,17]. These findings indicate that the common mechanism of arterial stiffness and retinopathy might be the accumulation of advanced glycation end products due to chronic glycemic exposure. The result of the association of other factors and diabetic retinopathy found in this study was similar with that which we have previously reported. HbA1c and hypertension were not significantly associated with diabetic retinopathy. UKPDS showed the development of retinopathy to be associated with long-term glycemic exposure and hypertension [16-18]. The duration of diabetes was found to be associated with diabetic retinopathy in this study. However, HbA1c is a marker for short-term glycemic control (1 or 2 months) only; thus the effects of glycemic control on diabetic retinopathy might, therefore, not be evident in this study. In addition, BP was not measured before the onset of diabetic retinopathy in our study, so the effects of hypertension might not be apparent. These results could be a consequence of the cross-sectional study design.

The duration of diabetes determined baPWV, but not AI, in this study. In addition, PWV was increased in patients with diabetes, but this is not associated with AI [3]. In this study, the correlation between baPWV and AI was weak. These results indicated that chronic hyperglycemia maynot be associated with increasing AI. On the other hand, diabetic retinopathy is associated with chronic hyperglycemia. Therefore, the result that AI was not associated with diabetic retinopathy in this study may be due to AI not being associated with the duration of diabetes.

AI was correlated with height, heart rate, SBP. On the other hand, baPWV was correlated with age, SBP, and heart rate [10]. In addition, AI was influenced by vasoactive drugs independently of PWV [19]. Thus, baPWV and AI were not interchangeable as markers of arterial stiffness. AI has stronger potential for use in endothelial function testing rather than as a marker of arterial stiffness compared with PWV [1]. These differences between PWV and AI may have includend the results of the study.

Insulin sensitivity reportedly influences AI [20], but was not examined in this study. Differences in insulin sensitivity may influenced the result of this study. PWV and AI are both strongly related to age, but the age-related changes in PWV and AI are non linear, with AI increasing more in younger individuals [11,12]. The age of the patients in study was not as advanced. These factors may be affected the result of our study.

A positive association between AI and diabetic retinopathy has been previously reported [21]. In that study, subjects with macroangiopathy were included. Diabetic retinopathy is also reportedly associated with cardiovascular disease [22]. Both AI and PWV are also associated with macroangiopathy [23,24]. These associations may have influenced the reported result. In this study, to avoid the association between arterial stiffness and macroangiopathy, patients with macroangiopathy were excluded. In addition, data of height and heart rate were not included. Height and heart rate are reportedly associated with PWV and AI [10], so these unincorporated data may have influenced their result. Another possibility is that the devices in each study were different. A well-designed study will be needed to clearly elucidate the association between AI and diabetic retinopathy, and to identify the relevant features with utility for clinical practice.

## Conclusion

PWV and AI are reported markers of arterial stiffness, but differing results have been obtained for diabetic retinopathy. The clinical significance of PWV and AI appears to be different in patients with diabetes.

## Competing interests

Omron Colin Corporation supported this study by supplying the pulse wave analyzer.

## Authors' contributions

OO participated in the design of the study and drafted the manuscript. KH and TW carried out measurements of pulse wave velocity and augmentation index. JK performed statistical analyses. MK and HY contributed to the study design and revised the manuscript. RK participated in the study design and coordination. All authors read and approved the final manuscript.
